# Epigenetic Dysregulation in *MYCN*-Amplified Neuroblastoma

**DOI:** 10.3390/ijms242317085

**Published:** 2023-12-03

**Authors:** Soraya Epp, Shin Mei Chuah, Melinda Halasz

**Affiliations:** 1 Systems Biology Ireland, UCD School of Medicine, University College Dublin, D04 V1W8 Dublin, Ireland; soraya.epp@ucdconnect.ie (S.E.);; 2 Conway Institute of Biomolecular and Biomedical Research, University College Dublin, D04 V1W8 Dublin, Ireland

**Keywords:** neuroblastoma, MYCN, epigenetics

## Abstract

Neuroblastoma (NB), a childhood cancer arising from the neural crest, poses significant clinical challenges, particularly in cases featuring amplification of the *MYCN* oncogene. Epigenetic factors play a pivotal role in normal neural crest and NB development, influencing gene expression patterns critical for tumorigenesis. This review delves into the multifaceted interplay between MYCN and known epigenetic modifications during NB genesis, shedding light on the intricate regulatory networks underlying the disease. We provide an extensive survey of known epigenetic mechanisms, encompassing DNA methylation, histone modifications, non-coding RNAs, super-enhancers (SEs), bromodomains (BET), and chromatin modifiers in *MYCN*-amplified (MNA) NB. These epigenetic changes collectively contribute to the dysregulated gene expression landscape observed in MNA NB. Furthermore, we review emerging therapeutic strategies targeting epigenetic regulators, including histone deacetylase inhibitors (HDACi), histone methyltransferase inhibitors (HMTi), and DNA methyltransferase inhibitors (DNMTi). We also discuss and summarize current drugs in preclinical and clinical trials, offering insights into their potential for improving outcomes for MNA NB patients.

## 1. Introduction

Neuroblastoma (NB), an embryonal tumor arising from the peripheral sympathetic nervous system, represents a challenging clinical entity characterized by heterogeneity in clinical presentation, prognosis, and treatment response [1]. Among the various genetic alterations observed in NB, amplification of the *MYCN* oncogene has emerged as an adverse prognostic genetic event. *MYCN*-amplification (MNA) occurs in approximately 20% of NB cases. MNA is associated with an aggressive phenotype, treatment resistance, and poor prognosis [1,2]. Treatment of high-risk NB involves intense genotoxic multiagent chemo- and radiation therapy [3,4]. About half of the high-risk patients relapse. Tragically, for survivors, genotoxic therapies can cause severe treatment-related morbidities, including hearing loss, infertility, endocrine deficiencies, and secondary cancers [5].

In recent years, the field of epigenetics has garnered significant attention in cancer research, unraveling the intricate regulatory mechanisms that dictate gene expression patterns beyond the DNA sequence itself. Epigenetic modifications, including DNA methylation, histone modifications, and chromatin remodeling, play fundamental roles in cellular development, differentiation, and disease pathogenesis [6]. Moreover, aberrant epigenetic alterations have been increasingly implicated in various cancers, providing a promising avenue for targeted therapeutic interventions [7].

In the context of MNA NB, understanding the interplay between genetic and epigenetic factors is of utmost importance. Epigenetic modifications have been recognized as crucial determinants of MYCN expression levels during neural crest development and in NB development [8]. Epigenetic modifications can indeed modulate the accessibility of transcriptional machinery to the *MYCN* locus, consequently influencing downstream gene networks involved in cell proliferation, differentiation, and apoptosis. Moreover, MYCN itself can regulate epigenetic processes in MNA NB. In this review, we summarize the latest advances in understanding the epigenetic dysregulation in MNA NB, and we highlight new therapeutic avenues targeting epigenetics for this high-risk pediatric malignancy.

## 2. Epigenetic Regulation of MYCN Expression during Neural Crest Development

### 2.1. Neural Crest (NC) and NB Development

**Figure 1:**
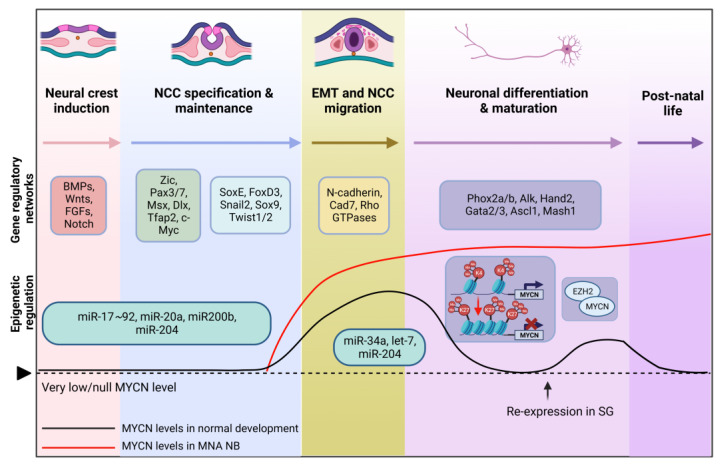
Neural crest development. The figure highlights key gene regulatory networks shaping neural crest induction, NCC specification, migration, and differentiation. The graph illustrates varying MYCN protein expression levels during normal NCC development (black line) and in MNA NB (red line). *MYCN* amplification might occur during early NCC migration or sympathetic lineage specification, leading to NB formation. Epigenetic mechanisms (miRNAs, histone methylation, and chromatin remodeling) regulating MYCN expression are also captured [9,10,11,12,13,14,15,16,17,18,19,20,21,22,23,24,25,26,27,28,29,30,31,32,33,34,35,36,37]. (SG: sympathetic ganglia). Created with Biorender.com, accessed on 3 November 2023.

Neural crest cells (NCC) arise at the neural plate border during gastrulation and neurulation in the third week of human development. NCCs undergo a series of developmental processes, including specification, migration, differentiation, and maturation. These processes are tightly regulated by gene regulatory networks involving various transcription factors (TFs) activated by bone morphogenetic proteins (BMPs), fibroblast growth factors (FGFs), wingless-type (WNT), and Notch signaling pathways (Figure 1) [9,10,11,12]. These inductive signals from the surrounding tissues activate the expression of NCC specifiers in the neural folds and define cells with NC identity. During neurulation, NCCs undergo epithelial-to-mesenchymal transition (EMT) and migrate extensively to distant locations in the embryo [13]. Throughout their migration, NCCs continuously respond to environmental cues, giving rise to diverse lineages and cell types that contribute to specific organ systems, such as the peripheral nervous system [14,15]. Notably, NC-derived cells persist into adulthood and possess stemness properties, potentially playing important roles not only in tissue regeneration but also in the initiation of cancer [16]. Information regarding the development of the neural crest is comprehensively outlined in multiple review papers [13,15,17,18].

**Figure 2:**
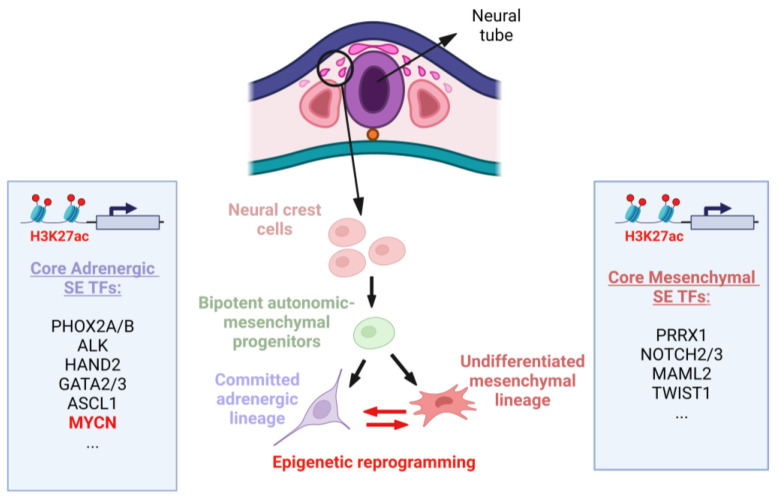
Early stages of NB development from NCCs. NB tumors exhibit two super-enhancer (SE)-associated cell types: committed adrenergic (ADRN) and undifferentiated mesenchymal (MES) cell types, originating from bipotent progenitors. These cells can spontaneously interconvert into one another via epigenetic mechanisms. This bidirectional conversion is controlled by core regulatory circuitries, where core TFs bind to their own SEs and each other’s SEs, creating feed-forward transcriptional loops that underlie lineage identity. The main core ADRN and MES SE-associated TFs are listed in the blue boxes. SEs were identified using genome-wide profiling of histone H3 lysine 27 acetylation (H3K27ac) enhancer signature in NB tumors and cell lines [18,25]. Created with Biorender.com, accessed on 5 October 2023.

NB specifically originates from the sympathetic lineage of NCCs [1,17]. Instead of differentiating into mature sympathetic neurons, NB cells maintain a more primitive and undifferentiated state, contributing to tumor heterogeneity and resistance to differentiation-inducing therapies [2,19]. Two distinct phenotypic subpopulations can be identified: adrenergic-type (ADRN) cells committed to the sympathetic lineage and undifferentiated mesenchymal-type (MES) cells resembling NC-derived precursors (Figure 2). These subpopulations exhibit different gene expression signatures and can interconvert via epigenetic reprogramming, mimicking cells from different stages of lineage differentiation [17,18]. 

### 2.2. MYCN Levels during Neural Crest and MNA NB Development

One of the master regulators of NCC development is the MYCN TF. The expression levels of MYCN vary during NCC development (Figure 1). During early nervous system development, MYCN is excluded from NCC stem cells, and *MYC*, a paralogue of *MYCN*, is transcribed, maintaining the multipotent NCC progenitors [17]. Later, MYCN is expressed in the migratory and post-migratory NCCs. MYCN initially exhibits a uniform expression pattern in migrating NCCs, but then its expression is subsequently downregulated to very low levels before the cells gather to form the ganglia, with later re-expression in NC-derived lineages [26]. After migration, MYCN expression becomes specifically limited to cells that are actively engaged in neuronal differentiation. This suggests that MYCN expression is a critical factor that influences whether NCCs become neurons or adopt a non-neuronal fate [27]. Indeed, its re-expression is associated with the maintenance of neural fate and the promotion of differentiation and functionality of sympathetic neurons [17,18,20,21]. Notably, MYCN is essential for inhibiting neuronal differentiation during neurogenesis, with limited impact on progenitor cell apoptosis [3,22]. MYCN was found to be re-expressed in differentiating sympathetic ganglia (SG) following the initiation of expression of lineage-determining transcription factors (TF) such as PHOX2B and HAND2 [23,24]. However, the role of MYCN extends beyond migration and differentiation, as it also influences cell growth and apoptosis during murine sympathoadrenal (SA) development. After birth, MYCN is not expressed in the SG and is nearly entirely absent in all tissues of adult mice [24,25]. 

Aberrations in MYCN expression influence NC cell fate decisions, impairing the normal processes of neuronal differentiation and migration. In mice, loss of *MYCN* results in decreased size of the entire nervous system, along with a reduction in the number of mature neurons in the spinal ganglia [17]. Notably, enforced expression of MYCN in mouse migrating NCCs leads to the development of NB-like tumors, while its overexpression in SA progenitors is not sufficient for neoplasia [30,38]. Patients with low-risk NB do not exhibit MNA nor progression to more metastatic and invasive disease, indicating that MNA serves as an early and potentially initiating event in the development of high-risk NB tumors [4]. 

The importance of MYCN in NB development is supported by findings from genetically engineered animal models of NB. Transgenic mice expressing perinatal *MYCN* under the tyrosine hydroxylase (*TH*) promoter, which is active in early migrating sympathetic precursors, spontaneously develop morphologically and phenotypically similar tumors to high-risk NB in humans [20,39,40]. The development of NB in these models is dependent on MYCN dosage, as MYCN alone is sufficient to drive NB formation [19,21]. In zebrafish models, ectopic MYCN expression in sympathetic precursor cells hinders the development of chromaffin cells, leading to NB formation [21,41]. Altogether, these findings suggest that the early exposure of NCCs to elevated MYCN levels is what could play a significant role in triggering the onset of MNA NB.

### 2.3. Epigenetic Regulation of MYCN Expression during NC Development and Implications for MNA NB

Several epigenetic mechanisms involved in NC development are known to be disrupted in NB genesis. 

MicroRNAs (miRNAs) play important regulatory roles during NC development, where they work in coordination with other regulatory mechanisms to fine-tune gene expression and ensure the proper development of NCCs into diverse cell types, including neurons. MYCN influences the expression of multiple miRNAs and is itself subject to miRNA regulation. This feedback mechanism is disrupted in NB, where upregulated levels of MYCN coincide with altered expression of specific miRNA clusters [28,29]. In normal NC development, miR-200b, miR-17~92, miR-20a, and miR-204 are known to be involved in NC induction/specification, while miR-34a, let-7, and miR-204 play roles in NCC EMT and migration (Figure 1, Table 1) [42]. Together, they contribute to NCC identity by inhibiting specific growth signaling pathways, eventually regulating key TFs such as MYCN. In the context of MNA NB, let-7, miR-34a, miR-200b, and miR-204 are tumor suppressor miRNAs found to be downregulated, while miR-17-92 and miR-20a are oncogenic miRNAs that are upregulated [29,31,32]. As MYCN levels need to be tightly controlled to ensure proper development, dysregulation in the MYCN-miRNA interplay could thereby impact the balance between cell proliferation and differentiation of NCCs, potentially leading to tumorigenesis. 

Investigation of histone modifications at the *MYCN* gene between normal and NB cells during mouse sympathetic cervical ganglia (SCG) development (E11.5) revealed a failure in the shift of histone marks from active H3K4me3 (trimethylation of lysine residue K4 on histone H3) to repressive H3K27me3 (trimethylation of lysine residue K27 on histone H3) [34]. Indeed, as mentioned earlier, MYCN expression is gradually downregulated to low levels for differentiation in normal development, consistent with a change to repressive H3K27me3 marks. Therefore, a lack of proper epigenetic repression during SCG development could lead to sustained expression of MYCN, potentially resulting in uncontrolled cell proliferation and NB formation [34].

Enhancer of Zeste Homolog 2 (EZH2), a core catalytic subunit of the Polycomb Repressive Complex 2 (PRC2), is a histone methyltransferase (HMT) that represses transcription via H3K27me3 [43]. The comparison of transcriptomes between wild-type (WT) and early-stage cancer cells from *TH-MYCN* mice (at E13.5) demonstrated a significant downregulation of PRC2 target genes, indicated by an increase in H3K27me3 levels around their promoters. Specific inhibition of EZH2 reversed the repression of target genes and eventually suppressed in vivo tumor growth in *TH-MYCN* mice [35,36]. Moreover, a physical interaction between MYCN and EZH2 was reported, suggesting recruitment of PRC2 by MYCN at specific genomic loci for EZH2-mediated epigenetic silencing of target genes [35,37] (Table 1). 

Collectively, these findings indicate that MNA NB initiation occurs during early migration or sympathetic lineage specification and necessitates continued perinatal expression of MYCN for tumorigenesis [20]. Persistent expression of MYCN in maturing sympathetic precursor cells can inhibit apoptotic signaling and sustain proliferation, thereby promoting NB development [21].

## 3. Altered Epigenetic Mechanisms in MNA Neuroblastoma

MYCN, in addition to its classical function as a TF, also plays a critical role in tumorigenesis via epigenetic regulation. In fact, MYCN has been shown to control multiple epigenetic mechanisms, including DNA methylation, histone modifications, miRNAs, and chromatin remodeling by directly regulating the transcription of epigenetic modifiers or via protein–protein interactions. The interplay between MYCN and chromatin-modifying mechanisms strongly influences disease progression and metastasis, highlighting these processes as key therapeutic vulnerabilities in MNA NB [44,45].

### 3.1. DNA Methylation in MNA NB

Methylation of DNA is one of several epigenetic mechanisms that cells use to alter gene expression. CpG islands (CGIs) are specific genomic regions characterized by a high frequency of cytosine–guanine (CpG) dinucleotides. These regions are often associated with gene promoters, transcription start sites, and first exons. DNA methylation at CpG islands is a crucial epigenetic mechanism implicated in cell fate determination during development, generally leading to gene silencing [45]. 

MYCN has been shown to bind to numerous promoters and CpG islands (CGIs) in NB, suggesting direct control of potential tumor suppressor genes via DNA methylation [6,44]. Specifically, methylation of caspase 8 (*CASP8*) and members of the Ras association domain family (*RASSF*) and protocadherin beta cluster (*PCDHB*) families have been extensively studied and linked to MNA [46,47,48,49]. Indeed, loss of *CASP8* by methylation is believed to allow for unhinged cell proliferation and therefore contribute to MNA NB tumorigenesis [49]. However, the correlation between *CASP8* methylation and MNA has been questioned by other groups [46,50]. 

Moreover, MYCN single-copy and MNA NB cell lines exhibit distinct promoter methylation patterns for genes like histone H3.1 (*HIST1H3C*) and acetyl-CoA synthetase short-chain family member 3 (*ACSS3*). Specifically, *HIST1H3C* methylation is associated with both overall and event-free survival [51]. The exception to the methylation pattern includes the hypomethylation of nuclear receptor 4A3 (*NR4A3*) exon 3 in MNA cells, which may be associated with a better prognosis [52], and the dual role of *CD44* methylation in both MNA and non-MNA NB [53,54,55]. 

Various studies suggest a CpG island methylator phenotype (CIMP) in NB, which is linked to poor survival. Both Japanese and German studies showed that MNA is linked to the CIMP+ phenotype [48,56], where among others, methylation levels of *PCDHB* CGIs defined the CIMP+ phenotype. Giwa et al., via differential methylation analysis of MNA versus non-MNA NB (TARGET data), identified 663 differentially methylated CpGs and 14 highly methylated genes associated with MNA in NB, suggesting that MNA alters the methylation landscape in NB and that this landscape differs from that in non-MNA NB [50]. In addition, Lalchungnunga et al. performed a genome-wide methylation analysis of the TARGET study and identified five distinct DNA-methylation-based molecular subgroups, where one subgroup is strongly associated with MNA [57]. The differentially methylated regions unique to the MNA subgroup contain 291 candidate genes, including *TERT* (cg11625005). In addition, MNA NB showed higher TERT expression compared to non-MNA NB.

Overall, DNA methylation is a critical epigenetic mechanism involved in the development of MNA NB (Table 2). However, the relationship between MYCN and DNA methylation in NB, along with the underlying molecular mechanisms, remains largely unexplored.

### 3.2. Histone Modifications in MNA NB 

Histone modifications, such as methylation, acetylation, phosphorylation, and sumoylation of histones, can influence the accessibility of DNA to the transcriptional machinery. Histone methylation can either activate or repress gene transcription, depending on which histone and which amino acid residue is methylated. For instance, methylation of histone H3 at lysine 4 (H3K4) is associated with gene activation, while methylation at H3K9 and H3K27 is linked to gene repression. Conversely, the addition of acetyl groups to histone tails generally results in a more open chromatin structure, facilitating increased gene transcription and is typically associated with gene activation. On the other hand, deacetylation is a hallmark of gene silencing.

#### 3.2.1. Histone Acetylation

MYCN has been shown to impact histone acetylation, the marker of transcriptional activation [68]. Importantly, MYCN can recruit histone acetyltransferases (HAT) to maintain chromosome acetylation and thus enhance transcription, particularly of genes involved in cell cycle progression and proliferation. The HAT E1A-binding protein (EP300) plays a critical role in establishing H3K27ac marks at super-enhancers in high-risk NB. EP300 expression correlates with poor prognosis of NB patients and promotes cell proliferation in MNA NB cell lines (Table 3) [69]. EP300 regulates enhancers via interactions with a TF part of the ADRN lineage-defining core regulatory circuit (CRC) in NB, TFAP2β, and is essential for high-risk NB growth. Interestingly, EP300 interacts with MYCN and can modulate its stability via simultaneous regulation of its acetylation and ubiquitylation on Lys 199 [70]. Loss of *EP300* leads to a global loss of H3K27ac marks and loss of MYCN protein expression [69].

MYCN is known to upregulate histone deacetylases (HDACs), including HDAC1/2/5 and sirtuin-1 (SIRT1), to promote gene repression and oncogenesis in both NB cell and mouse models [71,72,73]. Indeed, MYCN upregulates HDAC2 to repress the tumor suppressor miR-183 [74]. Upregulation of HDAC5 results in the transcriptional repression of tetraspanin *CD9*, which contributes to the invasion and metastasis of MNA NB [75]. Upregulation of SIRT1 also increases the protein stability of MYCN. SIRT1 binds to the Myc homology box I domain of MYCN, leading to MYCN phosphorylation and stabilization [72]. MYCN in complex with MIZ1 and SP1 recruits HDAC1 to the tropomyosin receptor kinase A (*NTRK1*) promoter and downregulates TrkA expression, which is usually associated with spontaneous regression of NB [73]. 

**Table 3:** Regulators of histone marks in MNA NB.

Gene	Role	Methylation/Acetylation in MNA NB	Expression in MNA NB	Citation
*EP300*	HAT	H3K27 acetylation	Increased	[69,70]
*HDAC2*	HDAC	Deacetylates *MIR183* promoter	Increased	[74]
*HDAC5*	HDAC	Deacetylates *CD9*	Increased	[75]
*SIRT1*	HDAC	Deacetylation of tumor suppressors	Increased	[72]
*HDAC1*	HDAC	Deacetylates *NTRK1* promoter	Unknown	[73]
*DOT1L*	HMT	H3K79 methylation	Increased	[76]
*EZH2*	Catalytic subunit of PRC2 complex	H3K27 trimethylation	Increased	[37,55]
*KDM4B*	Histone demethylase	H3K9me3/me2 demethylation	Increased	[77]
*PRMT5*	HMT	H3R8 and H4R3 dimethylation	Increased	[78]
*WDR5*	Histone H3K4 presenter	H3K4 trimethylation	Increased	[79]

#### 3.2.2. Histone Methylation

EZH2, a subunit of PRC2, catalyzes the trimethylation of histone H3 lysine 27 (H3K27me3) at target promoters for gene silencing. MYCN directly interacts with EZH2 via the Myc homology box domain 3 [37]. MYCN was also found to interact with DOT1L, the sole known HMT catalyzing H3K79 methylation [76]. In fact, DOT1L-mediated H3K79 methylation at MYCN-responsive elements in target gene promoters is essential for MYCN-induced transcriptional activation in MNA NB cells. The authors further demonstrated that DOT1L is needed for MNA NB cell proliferation, and its suppression reduced NB tumor progression in xenograft tumor models [76]. 

Recently, KDM4B, a demethylase involved in histone modifications, was found to be highly expressed in MNA NB cells. KDM4B physically interacts with MYCN and prevents the accumulation of repressive H3K9me2/me3 marks at chromatin loci of target genes. Suppression of KDM4B leads to downregulation of major tumor genes, including miR-17-92a-1 cluster host gene (*MIR17HG*), M-phase inducer phosphatase 1 (*CDC25A*), SRY-box 2 (*SOX2*), KIT ligand (*KITLG*), versican (*VCAN*), and syndecan 1 (*SDC1*), while suppressing MYCN function in both NB cells and xenograft models [77]. Table 3 provides a summary of the regulators of histone methylation and acetylation linked to MNA NB.

#### 3.2.3. Histone Phosphorylation

MYCN is known to recruit Aurora-A to chromatin in the S-phase of the cell cycle, which in turn phosphorylates histone H3 at Ser 10 and promotes the incorporation of histone H3.3 into promoters, inhibiting accumulation of RNA:DNA hybrids (R-loops) [80]. 

### 3.3. Non-Coding RNAs and MNA NB

Non-coding RNAs (ncRNAs) are a diverse group of functional RNA molecules that do not code for proteins but play essential roles in regulating gene expression and contributing to various cellular processes, development, and disease. MYCN has been found to exert its influence not only on protein-coding genes but also on non-protein-coding genes like microRNAs (miRNA) and long non-coding RNAs (lncRNAs). The deregulation of these genes, driven by MYCN, significantly contributes to the development and progression of NB [44,81]. These ncRNAs can also directly target the 3’ untranslated region (3’UTR) of *MYCN* mRNA or act via indirect pathways to influence tumor progression [82].

#### 3.3.1. miRNAs

MicroRNAs (miRNAs) are small single-stranded RNA molecules that regulate gene expression by binding to target messenger RNA molecules [83]. MiRNAs can silence genes via processes like mRNA cleavage, destabilization of mRNAs, and inhibition of protein translation. 

In MNA NB, MYCN is known to be predominantly a repressor of tumor suppressor miRNA expression, although several miRNAs, including the miR-17-92 cluster, were found to correlate with MYCN expression [29,84]. 

Several key tumor suppressor miRNAs are shown to be repressed by MYCN in the context of MNA. Indeed, miR-184 or miR-542-5p overexpression in NB xenograft models inhibited tumor growth and metastasis [29,85]. MYCN was also shown to activate critical oncogenic miRNAs in NB and other solid tumors like non-small cell lung cancer and breast cancer [86,87]. The functionally well-studied miRNAs, such as miR-9 and miR-421, are directly activated by MYCN and are thought to contribute to NB tumorigenesis [88,89]. 

The interaction between MYCN and miRNAs is reciprocal, as miRNAs can directly target and regulate the oncogene. In particular, miR-34a is the most extensively studied miRNA that directly regulates MYCN expression and acts as a tumor suppressor by inducing NB cell apoptosis [31,64]. MiR-506-3p was also found to regulate MYCN expression via the zinc finger PLAGL2, which binds to the *MYCN* promoter region [82]. Other miRNAs such as miR-15a-5p, miR-15b-5p, miR-16-5p, miR-101, miR-628-3p, and let-7 inhibit tumor progression by negatively affecting MYCN expression [90,91]. Indeed, the LIN28B–let-7–MYCN axis is known to play a critical role in sustaining the oncogenic phenotype in NB [92]. LIN28B is a protein that binds small RNA and functions as a negative regulator of let-7 miRNA tumor suppressors. LIN28B can maintain high levels of *MYCN* mRNA and protein levels via downregulation of let-7 miRNA in MNA but also in non-MNA NB tumors [92,93].

The interaction between MYCN and different miRNAs varies depending on their specific characteristics, and the expression of miRNAs is thereby influenced by *MYCN* status [45]. Indeed, a study performed on NB patients identified a set of 38 differentially expressed miRNAs between the low/intermediate and the high-risk groups [94]. 

MYCN direct binding to miRNAs loci at the proximal region is thought to be the mechanism for activation or repression of miRNAs in NB, as observed with the miR-17-92 cluster for example [95]. As mentioned above, MYCN also has the ability to activate DNA methyltransferases (DNMTs), which can eventually modify the promoters of miRNAs in NB. For example, miR-34b-3p, miR-34b-5p, miR-34c-5p, and miR-124-2-3p are found to be downregulated by hypermethylation in a subset of high-risk NB patients [31,63,64]. Other miRNAs such as miR-106b-5, miR-202, miR-204, let-7, miR-17-5p, and miR-26a-5p have also been reported to be regulated by MYCN in NB [33,92,96,97]. 

Overall, miRNAs are considered great biomarker candidates, and their modulation holds potential as a novel therapeutic strategy for NB treatment. Several reviews extensively describe the role of miRNAs in NB and their involvement in prognosis, drug response, and resistance [28,83,98]. Specific miRNAs involved in MYCN regulation in MNA NB are summarized in Table 4. 

#### 3.3.2. LncRNAs

LncRNAs are a class of RNA molecules that are longer than 200 nucleotides which are often associated with chromatin-modifying complexes. Although very little is known about the role of lncRNAs in NB, a study identified “non-coding RNA expressed in aggressive neuroblastoma” (ncRAN; also known as small nucleolar RNA host gene 16, SNHG16) and lncUSMycN, as being associated with aggressive MNA NBs and poor prognosis (Table 5) [136,137]. *LncUSMycN* is located at the 2p 130-kb amplicon that is co-amplified with *MYCN* and is found to upregulate *MYCN* mRNA expression via binding to NonO protein. Knocking down of *lncUSMycN* expression was shown to reduce MYCN at the mRNA and protein level, thereby inhibiting MNA NB cell proliferation and tumor growth in in vitro and in vivo NB models, respectively [136]. In addition, overexpression of small nucleolar RNA host gene 1 (*SNHG1*) is also linked to MNA NB. SNHG1 might downregulate miR338-3p, which in turn leads to PLK4 overexpression, thus promoting proliferation, migration, and invasion [138]. Another lncRNA upregulated by MYCN is the “lncRNA highly expressed in neuroblastoma 1” (lncNB1), and it is linked to poor prognosis. LncNB1 upregulates E2F1 expression by binding RPL35, which leads to the transcription of *DEPDC1B* [139]. In addition, neuroblastoma differentiation marker 29 (NDM29) might be downregulated in MNA NB, and overexpression of NDM29 in MNA NB cells leads to differentiation [140].

Moreover, transcribed ultraconserved regions (T-UCRs), a novel subgroup of lncRNAs, have been discovered to be abnormally expressed in NB. These RNA transcripts are highly conserved across human, rat, and mouse genomes. Specific UCR expression profiles can be correlated with prognosis in high-risk patients and MNA status in NB [84].

Furthermore, the *MYCN* opposite-strand (MYCNOS) lncRNA, specifically MYCNOS-01 and 02, were found to regulate MYCN protein expression [141]. MYCNOS-02 interacts with specific binding partners like Ras GTPase-activating protein 1 (G3BP1) and recruits 11-zinc finger protein (CTCF) to the *MYCN* promoter, resulting in increased MYCN expression. This positive regulation of *MYCN* by MYCNOS-02 leads to suppressed differentiation and increased growth, invasion, and metastasis of NB cells. On the other hand, silencing of *MYCNOS-01* in MNA NB cell lines had a similar effect to MYCN reduction, suggesting the potential of these two lncRNAs as therapeutic targets [141,142].

#### 3.3.3. circRNAs

Circular RNAs (circRNAs) are a type of RNA molecule where the 3′ and 5′ ends are joined together in a closed-loop structure. This circular formation is produced via a mechanism known as back-splicing, where a downstream splice donor site is joined to an upstream splice acceptor site, resulting in a circRNA molecule. They have been implicated in various cellular processes, including acting as microRNA sponges and regulating gene expression. 

In a study characterizing circRNAs in NB cell lines, seven were located within MNA regions and were upregulated: hsa_circ_0003287, hsa_circ_0008083, hsa_circ_0052767, hsa_circ_0000978, hsa_circ_0117720, chr2:15467874|15567918, and hsa_circ_0008261 [41]. Importantly, has_circ_0000978 was also identified as a potential prognostic biomarker of acute myeloid leukemia resistance [143]. Investigating the global circRNA landscape in NB, Fuchs et al. highlighted a distinct circRNA expression profile in MNA NB [144]. They showed that MYCN suppresses circRNA expression via the DHX9 RNA helicase. In addition, circARID1A was identified as promoting the proliferation and survival of NB cells via its direct interaction with the KH-type splicing regulatory RNA-binding protein (KHSRP) [144]. Importantly, this circRNA is derived from the *ARID1* SWI/SNF tumor suppressor gene, which is almost always mutated in MNA NB, as described in Section 3.6. below [145,146]. Together, these studies highlight the importance of MYCN regulating circRNAs and their contribution to NB pathogenesis (Table 5). 

**Table 5:** LncRNAs and circRNAs in MNA NB.

Non-Coding RNA	Expression in MNA NB	Associated Function	Citation
ncRAN	Upregulated	Oncogenic	[137]
lncUSMycN	Co-amplified with *MYCN*	Oncogenic; upregulates *MYCN* mRNA expression via binding to NonO	[136]
SNHG1	Upregulated	Oncogenic; downregulates miR338-3p, leading to PLK4 overexpression	[138]
lncNB1	Upregulated	Upregulates E2F1 expression by binding RPL35, leading to transcription of *DEPDC1B*	[139]
NDM29	Downregulated	Induces differentiation of MNA NB cells when overexpressed	[140]
T-UCRs (uc.347, uc.350, uc.279, uc.460, uc.379, uc.446 and uc.364)	Upregulated	Oncogenic; involved in proliferation, apoptosis, and differentiation	[84]
MYCNOS-01	Co-amplified with *MYCN*	Oncogenic; regulates MYCN protein expression	[141,142]
MYCNOS-02	Co-amplified with *MYCN*	Oncogenic; interacts with G3BP1 and recruits CTCF to the *MYCN* promoter, thereby increasing MYCN expression. Suppresses differentiation and increases growth, invasion, and metastasis of NB cells	[141]
circ_0003287, circ_0008083, circ_0052767, circ_0000978, circ_0117720, chr2:15467874|15567918, circ_0008261	Upregulated	Oncogenic	[41]
circARID1A	Upregulated	Oncogenic; promotes proliferation and survival of NB cells via direct interaction with KHSRP	[144]

### 3.4. Super-Enhancers as Epigenetic Modifiers Regulating MYCN in NB

Enhancers are DNA sequences that can increase the transcriptional activity of nearby genes. Super enhancers (SEs) have a higher density of TF binding sites compared to typical enhancers. They are particularly associated with genes that control cell fate and cell-type-specific functions. The formation of SEs involves the binding of TFs, co-activators, and chromatin regulators to the DNA. This assembly of regulatory proteins leads to a highly accessible and active chromatin state, facilitating robust and specific gene expression, including oncogenes such as *MYCN* [147]. As mentioned earlier in Section 2.1, NB consists of two distinct cellular identities: committed ADRN and undifferentiated MES cell types [148]. These cells can switch between each other, leading to cellular and intra-tumoral heterogeneity. Both identities are shown to be associated with a distinct SE landscape. Most NB tumors show ADRN characteristics, but some exhibit MES features, especially in metastatic and relapsed cases associated with chemotherapy resistance (Figure 2) [148].

Indeed, the activation of MYCN in NB occurs via a process known as “enhancer hijacking”, where structural rearrangements or translocations bring distal regulatory elements into proximity with other genes, resulting in enhanced expression [149]. MNA in NB involves the co-amplification of proximal enhancers driven by the noradrenergic CRC or the loss of local gene regulatory elements due to ectopic enhancer hijacking [150]. MYCN also interacts with Twist-related protein 1 (TWIST1) and Achaete-scute family bHLH TF 1 (ASCL1) at enhancers to activate developmental genes crucial for MYCN-dependent proliferation and NB tumorigenesis [151,152]. 

Using genome-wide profiles of H3K27ac in primary and relapsed NB patients, Gartlgruber et al. identified four epigenetic subtypes driven by SEs. Specifically, three of these subtypes are characterized by ADRN-specific signatures that align with known clinical groups: MNA, non-MNA high-risk, and non-MNA low-risk NBs [153]. The fourth subtype with MES features is linked to relapsed NB. This study also identified highly specific modules of CRC TFs associated with particular subtypes. Notably, the MNA CRC TF subtype module includes MYCN, TWIST1, SRY-box TF 11 (SOX11), and T-box TF 2 (TBX2) [153]. 

To summarize, MNA allows stabilization of the CRC, thereby activating the MYCN transcriptional regulatory network and promoting an immature neuroblast cell state [148,154]. Disrupting the MYCN enhancer regulatory axis and targeting SEs holds promise as therapeutic strategies in NB, offering potential avenues to inhibit oncogenic transcription and inhibit tumor growth [154,155].

### 3.5. Bromodomains in MNA NB

The bromodomain and extraterminal (BET) subfamily consists of proteins that possess bromodomains, which recognize and bind to acetylated lysine residues on histones. These proteins, including BRD2, BRD3, BRD4, and BRDT, play important roles in regulating gene transcription and are involved in various cellular processes linked to chromatin remodeling. BRD4 is known to have two distinct enzymatic functions: kinase and HAT activity. Indeed, BRD4 plays a crucial role in regulating transcription via the phosphorylation of various interacting partners, including RNA polymerase II (RNAPII) and MYC. BRD4 also acetylates nucleosomes and influences chromatin architecture via its HAT activity [156]. Moreover, BRD4 serves as a significant regulatory factor for active enhancers and SEs throughout the genome. It has been demonstrated that BRD4 directly controls the *MYC* SE in acute myeloid leukemia cells, highlighting its critical role in SE regulation. In MNA NB, the elevated levels of MYCN promote increased histone acetylation, thereby creating more binding sites for bromodomain proteins. Due to their involvement in cancer-related processes, BET proteins have garnered significant attention as potential therapeutic targets. Several small molecule inhibitors targeting BRD4 specifically have been developed and tested in preclinical and clinical studies, showing promise as anti-tumor agents in NB [157,158,159].

### 3.6. Chromatin Remodeling Complexes in MNA NB

Chromatin remodeling complexes depend on ATP to mobilize and restructure selected nucleosomes. These multi-subunit protein complexes can be divided into four subfamilies: switch/sucrose non-fermentable (SWI/SNF), imitation switch (ISWI), chromodomain helicase DNA-binding (CHD), and inositol 80 (INO80) chromatin remodelers. These complexes are conserved across eukaryotes and are involved in dynamic changes of nucleosome architecture, allowing better or less access to DNA by various cellular machinery, including TFs and RNA polymerases. 

Mammalian SWI/SNF complexes are part of three subfamilies: canonical BAF, polybromo-associated BAF, and non-canonical BAF. Mutations or alterations in *SWI*/*SNF* subunits are often associated with cancer, including NB [145,146]. *ARID1A* encodes a subunit of the canonical BAF complex and is deleted on one allele in at least 87% of NB cases with loss of chromosome 1p. The chromosome 1p36 region is almost always missing in MNA NB, resulting in at least one-third of NB cases harboring haploinsufficiency for *ARID1A*. Using a transgenic *MYCN* zebrafish model of high-risk NB, researchers demonstrated that disrupting *ARID1A* enhances MYCN-induced cell proliferation in the SA lineage. In *ARID1A* homozygous mutant MNA NB cell lines, the differentiation status shifted from adrenergic to mesenchymal, resulting in increased invasiveness. This transition is orchestrated by modifications in enhancer-driven gene expression via the modification of binding sites for both BAF and PBAF complexes [146]. In line with those results, a more recent study investigated the role of SMARCE1, a BAF subunit, in MNA NB [160]. *SMARCE1* is located on chromosome 17q, which is frequently gained in NB and correlates with MNA and poor prognosis. High SMARCE1 expression is found to correlate with poor prognosis and is necessary for the proliferation and survival of MNA NB cells. Moreover, not only does MYCN directly upregulate *SMARCE1* transcription by binding to its promoter, but both proteins interact to regulate MYCN target genes. These results underscore the possibility for SMARCE1 to modify nucleosome structure and chromatin accessibility, aiding in the transcriptional regulation of *MYCN*. Altogether, targeting chromatin modifiers belonging to the SWI/SNF complexes could help restore normal gene expression patterns in MNA NB [160].

*CHD5* is also encoded at 1p36, and it is frequently lost or silenced in high-risk NB [161]. CHD5 is a chromodomain-helicase-DNA-binding protein that forms a nucleosome remodeling and deacetylation (NuRD) complex. It was shown that CHD5 induces the transcription of neuronal genes and represses the transcription of Polycomb target genes by maintaining H3K27me3 [162]. CHD5 function is required for neuronal differentiation, and loss of *CHD5* is often observed in high-risk NB, including MNA NB. However, this is irrespective of *MYCN* amplification [161].

## 4. Epigenetic Therapies for MNA NB

**Figure 3:**
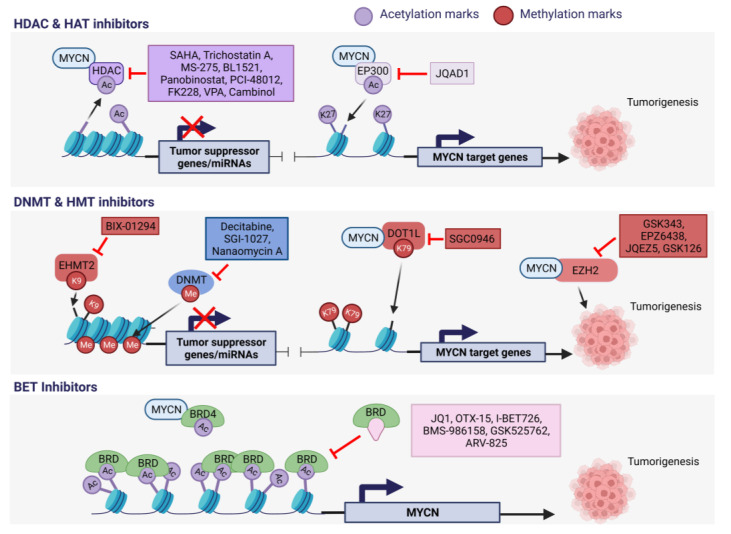
Epigenetic drug targets explored for MNA NB. In the realm of epigenetic regulation, inhibitors targeting three distinct epigenetic mechanisms: DNA methylation, histone methylation or acetylation, and epigenetic readers have been investigated in pre-clinical or clinical trials for MNA NB [35,55,69,72,164,165,166,167,168,169,171,173,174,175,177,178,180,181,182,183,184,185,186]. Created with Biorender.com, accessed on 3 November 2023.

To date, there are not many studies on epigenetic drug targets for MNA NB. So far, the major classes of epigenetic drugs that have shown promise in MNA NB are histone deacetylase inhibitors (HDACi) and BET inhibitors (BETi), mostly in preclinical studies. A comprehensive summary of the existing literature on epigenetic drugs and their corresponding targets in the context of MNA NB is provided in Table 6 and illustrated in Figure 3. 

### 4.1. HDAC and HAT Inhibitors in MNA NB

A compound screen performed by Krstic et al. revealed that NB cells are vulnerable to various classes of epigenetic regulators, and they showed that C646, a CBP/p300 HAT inhibitor, is specifically effective in reducing the viability of NB cells with MNA [163].

As already mentioned above, MNA in NB leads to the suppression of tumor suppressor genes via the recruitment of DNMTs and increased expression of HDACs. Promising preclinical studies using HDAC inhibitors in the *TH-MYCN* NB mouse model have been conducted. Indeed, a positive feedback loop between MYCN and the HDAC SIRT1 has been described, and treatment with the SIRT1 inhibitor cambinol was able to reduce tumorigenesis in the transgenic NB model [72]. Additionally, trichostatin A, another HDAC inhibitor, restored the expression of the differentiation protein transglutaminase 2 (TG2), usually repressed by MYCN in NB cells, resulting in reduced tumor volume in the same mouse model [164]. 

Other HDAC inhibitors, such as MS-275, BL1521, and vorinostat (SAHA), were shown to decrease the cell viability of several NB cell lines [165,166,167]. Specifically, SAHA treatment is more efficient on cell lines carrying MNA and is extensively studied in clinical trials alone or in combination with other drugs. It is the first HDACi that has been approved by the US Food and Drug Administration (FDA), on 6 October 2006, for the treatment of cutaneous T cell lymphoma [165,168]. Together with panobinostat, which is also FDA-approved, they have successfully completed phase II of clinical trials in NB patients [169]. 

Other drugs targeting HDAC1/2/8, which are associated with poor prognosis in NB, have sparked interest [170]. In fact, treatment with a small-molecule inhibitor of HDAC8 showed great inhibitory activity against NB growth in vitro and in vivo and enhanced retinoic-acid-mediated differentiation [170]. 

Romidepsin (FK228), an HDAC1/2i FDA-approved drug, has been shown to be specifically potent in MNA NB cells by increasing histone acetylation and thereby cell death via caspase-dependent apoptosis [171]. Further, the grainyhead-like 1 (*GRHL1*) gene was identified as an early response gene following HDACi treatment, which is usually repressed by MYCN via HDAC3. This TF, conserved throughout evolution, plays a crucial role in the development of the nervous system in *Drosophila* [172]. Importantly, high levels of GRHL1 in NB tumors correlate with event-free patient survival and favorable tumor biology [172].

One HAT inhibitor, JQAD1, has been recently investigated in pre-clinical trials on MNA NB cells [69]. JQAD1 is a proteolysis-targeting chimera (PROTAC) compound, developed to selectively degrade the HAT EP300. The role of EP300 in MNA NB is discussed in Section 3.2.1. The small innovative PROTAC degrader showcases a time-dependent depletion of EP300, resulting in rapid loss of MYCN expression and apoptosis of NB cells. Importantly, JQAD1 demonstrates low toxicity towards healthy cells while exerting growth-inhibitory effects of NB tumor xenografts [69].

Altogether, these findings highly suggest that targeting HDACs and, to a lesser extent, HATs, could be a potential approach to treat MNA NB.

### 4.2. HMT Inhibitors in MNA NB

As mentioned in Section 2.3 and Section 3.2.2, the HMT EZH2 is a protein of major importance during NB development. EZH2 levels are significantly higher in MNA cells than in non-MNA cells and this leads to the inactivation of a tumor suppressor program in NB [55]. Multiple studies have provided evidence supporting the therapeutic validity of S-adenosyl-methionine (SAM)-competitive EZH2 inhibitors in NB. SAM is a methyl donor for catalytic reactions of HMTs. Overall, inhibiting EZH2 directly via the occupation of the site for S-adenosyl-L-methionine (SAM) in the EZH2′s binding pocket inhibits MNA NB cell and tumor growth.

For example, GSK343 significantly reduced tumor growth in a MNA xenograft mouse model without noticeable toxicity [173]. Nonetheless, GSK343 is confined to pre-clinical use due to its inadequate pharmacokinetic characteristics [174]. Tazemetostat (EPZ6438) is an FDA-approved drug with high selectivity towards EZH2, which demonstrates a significant reduction in tumor weight in the *TH-MYCN *mouse model with no related adverse effect [35]. GSK126 also showed growth-inhibitory effects in in vitro and in vivo MNA NB models [55,175], but phase II clinical trials were halted due to insufficient efficacy in cancer patients [176]. Lastly, the effect of GSK126 was tested in combination with another EZH2 inhibitor, JQEZ5. The combination increased apoptosis in MNA cells, and JQEZ5 alone was able to significantly reduce tumor volume in an MNA xenograft mouse model [55]. 

In an immunology-related study, Seier and al. suggested exploring the use of H3K9 euchromatic histone-lysine methyltransferase (EHMT) inhibitors in combination with EZH2 inhibitors as an immunomodulation strategy for MNA NB treatment. Essentially, EHMT1 and EHMT2 were identified as key epigenetic factors involved in the malignancy of MNA NB and suppressors of interferon signaling. Inhibiting EHMT enhances the expression of Th1-chemokines, therefore facilitating T-cell infiltration into the tumor microenvironment and improving responsiveness to immune checkpoint blockade therapy [177]. Indeed, BIX-01294, an EHMT2 inhibitor, was shown to specifically decrease global H3K9me2 levels [178]. EHMT2 inhibition not only restrains the proliferation of NB cells but also triggers their apoptosis. BIX-01294 impedes MNA NB cell motility and invasion while concurrently suppressing the expression of the *MYCN* oncogene. Moreover, EHMT2 inhibition was found to synergize with doxorubicin, further hampering cell proliferation [178]. 

### 4.3. BET Inhibitors in MNA NB

Over the last few years, targeting BET family proteins has emerged as an eminent strategy in the treatment of cancers and other diseases. These proteins have a close association with the regulation of the *MYC* oncogene, making BET inhibitors effective in MYC-dependent cancers. By reducing the expression of oncoproteins like MYC, they effectively hinder the growth of malignant cells [179]. To date, targeting MYCN in MNA NB with BET inhibitors has been tested in preclinical and clinical studies. Via high-throughput pharmacological screening of more than 650 cancer cell lines, it was discovered that MNA strongly predicts the cytotoxic response to the prototypic BRD4 inhibitor, JQ1 [180]. JQ1 disrupts the recruitment of BRD4 to the *MYCN* canonical promoter by competitively binding to acetyl lysine sites, resulting in the downregulation of MYCN and its transcriptional outputs. In NB cells, JQ1 treatment leads to cell cycle arrest, apoptosis, and increased differentiation. Importantly, cell lines harboring MNA were found to be more sensitive to JQ1 than the non-amplified ones. Promising results were also observed in in vivo studies using MNA NB xenograft models, showing a significant decrease in tumor volume and increased overall survival without noticeable toxicity [180,181].

Building upon these findings, three JQ1 derivatives (OTX015, I-BET726, and BMS-986158) are currently under investigation, with BMS-986158 currently entering phase I in clinical trials (NCT03936465). OTX015, an orally available inhibitor of BRD2/3/4, demonstrates potent inhibition of tumor growth and increased overall survival in MNA NB cells and xenograft models. OTX015 has been found to exhibit specific activity against MYCN target genes, and this activity is correlated with high levels of MYCN expression and MNA in several NB cell lines [182]. 

Another small molecule BETi, GSK525762, exhibits anti-proliferative effects and cytotoxicity in NB cell models regardless of MYCN status, suggesting alternative mechanisms of action. A phase I study involving hematological and solid tumor cancers has shown therapeutic activity and tolerability of GSK525762, paving the way for potential translation to NB clinical studies [183]. However, the predictive value of MYCN overexpression for therapeutic response to GSK525762 remains unclear. 

Additionally, preclinical studies exploring combinations of BET inhibitors with other therapeutic agents, such as phosphoinositide 3-kinase (PI3K), cyclin-dependent kinase 7 (CDK7), mitogen-activated protein kinase (MEK), HDAC, and Aurora A inhibitors, have shown synergistic effects, and potential to overcome resistance, and reduce treatment-related toxicities [158,159,187,188]. These combinatorial approaches hold promise in maximizing treatment efficacy and addressing concerns regarding drug resistance and lack of specificity associated with genotoxic treatments.

### 4.4. DNA Methyltransferase Inhibitors in MNA NB

Only very few hypomethylating agents have been tested so far in preclinical and phase I clinical trials against NB. Decitabine and 5-azacytidine are DNMT inhibitors (DNMTi) primarily used in the treatment of myelodysplastic syndromes (MDS). Decitabine was found to epigenetically activate miR-34a when combined with retinoic acid, resulting in MYCN downregulation in acute myeloid leukemia patients and cultured cells [184]. This is particularly interesting since miR-34a is a tumor suppressor that is downregulated in the context of MNA NB [31,64]. Decitabine was part of three phase I clinical trials involving NB patients and is typically used in combination with chemotherapy to enhance its effectiveness. However, when combined with doxorubicin, it seemed that doses of decitabine that could have a substantial clinical impact were associated with poor tolerance [189].

Recently, two less toxic DNMTi, SGI-1027 and nanaomycin A, displayed enhanced NB cell death when combined with doxorubicin, compared to doxorubicin alone. Even though this effect was MYCN-independent, cell lines harboring MNA were at least 20 times more sensitive to the DNMT3b inhibitor nanaomycin A [185]. In line with this finding, retinoic acid treatment of MNA NB cells was shown to downregulate DNMT3b and upregulate miR-26a/b, a DNMT targeting miRNA normally repressed by MYCN [186]. Therefore, these results suggest that the downregulation of DNMTs leading to demethylation and reactivation of specific genes and miRNAs might offer new alternatives of specific treatment against MNA NB. 

A table showcasing the comprehensive range of epigenetic drugs currently being explored in preclinical and clinical trials for the treatment of MNA NB is summarized in Table 6.

**Table 6:** Drugs and associated epigenetic targets in the context of MNA NB.

Name	Drug Target in NB	Effect on MYCN in MNA NB	Clinical Status	Citation
ARV-825	BRD4 inhibitor	Downregulation	Pre-clinical	[190]
BIX-01294	EHMT2 (HMT) inhibitor	Downregulation	Pre-clinical	[178]
BL1521	Pan-HDAC inhibitor	Downregulation	Pre-clinical	[166,191]
BMS-986158	BRD4 inhibitor	Not specifically characterized	Pre-clinical and clinical Phase I: - NCT03936465 (recruiting)	[169]
Cambinol	SIRT1 (HDAC) inhibitor	Not specifically characterized	Pre-clinical	[72]
Decitabine (5-Azacytidine)	DNMT 1 inhibitor	Not specifically characterized	Pre-clinical and clinical Phase I: - NCT00075634 (completed) - NCT01241162 (completed) - NCT03236857 (completed)	[184,192]
Entinostat (MS-275)	HDAC I inhibitor	Downregulation	Pre-clinical	[167]
GSK343, Tazemetostat (EPZ6438), JQEZ5, GSK126	EZH2 inhibitors	Downregulation (GSK343), others not specifically characterized	Pre-clinical and clinical Phase II (EPZ6439): - NCT03155620 (recruiting)	[35,55,173,175,193]
I-BET726 (GSK726)	BRD4 inhibitor	Downregulation	Pre-clinical	[194]
JQ1/OTX-015	BRD4 inhibitor	Downregulation	Pre-clinical	[159]
JQAD1	EP300 (HAT) inhibitor	Downregulation	Pre-clinical	[69]
Molibresib (GSK525762, I-BET762)	BRD2/3/4	Downregulation	Pre-clinical and clinical Phase I: - NCT01587703 (completed)	[195]
Panobinostat	Pan-HDAC inhibitor	Downregulation	Pre-clinical and clinical Phase II: - NCT04897880 (terminated)	[159]
PCI-48012	HDAC 8 inhibitor	Downregulation	Pre-clinical	[170]
Romidepsin (FK228)	HDAC I inhibitor	Not specifically characterized	Pre-clinical	[171]
SGC0946	DOT1L (HMT) inhibitor	Unknown	Pre-clinical	[76]
SGI-1027, Nanaomycin A	DNMT1/3 inhibitors	Not specifically characterized	Pre-clinical	[185]
Trichostatin A	HDAC I/II inhibitor	Downregulation	Pre-clinical	[164]
Valproic acid (VPA)	HDAC I inhibitor	Downregulation	Pre-clinical and clinical Phase I: - NCT01204450 (terminated)	[196]
Vorinostat (SAHA)	HDAC I inhibitor	Downregulation	Pre-clinical and clinical Phase I: - NCT00217412 (completed) - NCT01132911 (completed) - NCT01019850 (completed) - NCT01208454 (completed) - NCT03332667 (active) - NCT04308330 (recruiting) Phase II: - NCT02035137 (completed) - NCT03561259 (recruiting) - NCT02559778 (recruiting)	[165]

## 5. Conclusions

Half of the children with high-risk NB do not respond to current treatments and relapse. Genotoxic chemotherapy is the mainstay of therapy for high-risk NB as we lack targeted treatments for this aggressive pediatric malignancy. Therefore, better and less toxic treatments are desperately needed. Targeting epigenetic machinery is a promising approach as MYCN regulates epigenetic processes. Epigenetic dysregulation is a hallmark of cancer, including NB, and contributes to NB development, tumor growth, and progression. By specifically targeting the aberrant epigenetic modifications, epigenetic drugs have the potential to restore normal gene expression patterns, inhibit tumor growth, and overcome drug resistance. The major classes of epigenetic drugs that have so far shown promise in MNA NB are histone deacetylase inhibitors and BET inhibitors, with a more recent focus on drug combination therapies.

Epigenetic drugs offer the advantage of potentially greater specificity, targeting cancer cells while minimizing damage to normal cells. They can potentially enhance the efficacy of conventional chemotherapy and reduce the reliance on highly toxic agents, thereby mitigating some of the side effects associated with genotoxic treatments in young patients. Additionally, exploring epigenetic drugs in high-risk NB may provide alternative treatment options for patients who do not respond well to standard chemotherapy or who experience disease relapse. However, further research and clinical trials are needed to establish their safety, efficacy, and optimal use in the context of high-risk NB. Efforts are also being made to identify biomarkers that can help predict patient response to epigenetic drugs and guide personalized treatment decisions. 

Moving forward, further research into novel treatment targets involving epigenetic-related molecules and their interactions with MYCN holds promise. By focusing on these avenues, we can advance our understanding and potentially discover more effective therapies for *MYCN*-driven NB. 

## Figures and Tables

**Table 1:** Epigenetic mechanisms in normal NC and in MNA NB development.

Epigenetic Mechanism	Role in Normal NC Development	Aberration in MNA NB	Citation
miR-200b	NC induction/specification	Tumor suppressor; downregulated	[28,32,42]
miR-17~92	NC induction/specification	Oncogenic; upregulated	[28,29,42]
miR-20a	NC induction/specification	Oncogenic; upregulated	[28,29,42]
miR-204	NC induction/specification, NCC EMT/migration	Tumor suppressor; downregulated	[28,42]
miR-34a	NCC EMT/migration	Tumor suppressor; downregulated	[28,29,31,32,42]
let-7	NCC EMT/migration	Tumor suppressor; downregulated	[28,29,42]
Histone modifications of the *MYCN* gene	Shift from active H3K4me3 to repressive H3K27me3 mark for *MYCN* downregulation during neuronal differentiation/maturation	The active H3K4me3 mark is kept; *MYCN* expression is sustained	[34]
EZH2	Controls the expression of genes crucial for neuronal differentiation/maturation via histone methylation, H3K27me3 is associated with gene repression	Recruitment of PRC2 by MYCN for EZH2-mediated epigenetic silencing	[35,37]

**Table 2:** DNA methylation patterns linked to MNA NB.

Gene	Role	Methylation Status in MNA NB	Expression in MNA NB	Citation
*ABCB1*, *CACNA1G*, *CD44*, *DUSP23*, *PRDM2*, *RBP1*, *CHD5*, *NTRK1*, *KRT19*, *PRPH*, *CNR1*, *QPCT*, *ASIC2*, *RGS5*	Involved in NB-relevant aberrant methylation	Hypermethylation	Decreased	[48]
*CASP8*	Cell apoptosis	Hypermethylation	Decreased	[46,47,49,58]
*CASR*	Calcium-sensing receptor	Hypermethylation	Decreased	[59]
*CD44*	Glycoprotein involved in cell–cell interactions, adhesion, and migration	Hypermethylation	Silenced	[54,60]
*CXCR4*, *GAL*, *LRRN1*, *ODC1*, *TWIST1*, *WHSC1* *DDX43*, *PRAME*, *TEX14*, *TMEM108*, *NEK2*, *NPY*	Involved in biology of aggressive NB	Hypomethylation	Increased	[48]
*DNAJC15*, *NTRK1*, *PYCARD*	Candidate biomarker genes	Hypermethylation	Decreased	[61]
*DUSP2*, *TP73*, *JAK2*, *MGMT*, *HPN*, *RB1*, *TDGF1*	Relevant roles in cancer biology	Hypermethylation	Increased/Decreased	[62]
*MIR34B*, *MIR34C MIR124-2*	MiR-34b-3p, miR-34b-5p, miR-34c-5p, and miR-124-2-3p are tumor suppressors	Hypermethylation	Decreased	[31,63,64]
*NR4A3*	Critical gene for neuronal development	Hypomethylation	Decreased	[52]
*NXPH1*, *SOX2-OT*, *DLX5*, *TFAP2D*, *CAVIN3*, *VAX2*, *TERT*, *HHEX*, *KRT19*, *RNF207*, *MIRLET7BHG*, *CHRNE*, *DLX6-AS1*, *TMCO3*	14 highly methylated genes in MNA NB	Hypermethylation		[50]
*PCDHB family*	Cell–cell neural connection	Methylation	Unknown	[48]
*RASSF family*	Tumor suppressor proteins	Hypermethylation	Decreased/absent	[47]
*TFAP2B*	Transcription factor, expression associated with low-risk NB	Methylation	Decreased	[65]
*ZAR1*	Ovary-specific maternal factor	Hypermethylation	Increased	[66]
*ZNF206*	Transcription factor regulating embryonic stem cell gene expression and differentiation	Hypomethylation	Unknown	[67]
291 genes	Candidate differentially methylated regions unique to NB subgroup associated with MNA			[57]

**Table 4:** MYCN-related miRNAs and their function in NB.

miRNA	Expression in MNA NB	Associated Function	Citation
let-7	Downregulated	Tumor suppressor. Controls sympathetic neurogenesis; promotes neuronal differentiation	[29,99,100]
miR-7	Upregulated	Oncogenic. Involved in cortical development and embryonic stem cell differentiation	[101,102]
miR-9	Upregulated	Oncogenic. Regulates neurogenesis (neuronal migration and differentiation)	[88,103]
miR-15a-5p	Downregulated	Tumor suppressor. Antiangiogenic in the brain	[90,104]
miR-15b-5p	Downregulated	Tumor suppressor	[90]
miR-16-5p	Downregulated	Tumor suppressor. Antiangiogenic in the brain	[90,104]
miR-17-5p	Upregulated	Oncogenic. Master regulator of neurogenesis in both developmental and adult brains	[29,105]
miR-19a-3p	Upregulated	Oncogenic. Enriched in NPCs and downregulated during neuronal development in the adult hippocampus	[29,106]
miR-19b-3p	Downregulated	Tumor suppressor. Enriched in NPCs and downregulated during neuronal development in the adult hippocampus	[106,107]
miR-20a-5p	Upregulated	Oncogenic. Inhibits cyclin D1 level, involved in differentiation and proliferation of cortical progenitors	[29,108]
miR-26a	Downregulated	Tumor suppressor. Regulates neural differentiation	[92,109]
miR-29 (miR-29a-3p, miR-29b-3p, miR-29c)	Downregulated	Tumor suppressor. Inhibits apoptotic neural death by targeting the proapoptotic protein BCL2	[49,101,110]
miR-34a	Downregulated	Tumor suppressor. Regulates neural stem/progenitor cell differentiation	[31,64,111]
miR-34c-5p	Upregulated/Downregulated	Oncogenic/Tumor suppressor. Regulates neural stem/progenitor cell differentiation	[29,31,64,112]
miR-93-5p	Downregulated	Tumor suppressor. Maintenance/proliferation/differentiation of NSCs; downregulated in mature neurons	[29,113]
miR-98-5p	Downregulated	Tumor suppressor	[114]
miR-101-3p	Downregulated	Tumor suppressor. Involved in neuronal plasticity	[91,115]
miR-106a-5p	Upregulated	Oncogenic	[116]
miR-106b-5	Upregulated	Oncogenic. Involved in NSC proliferation and differentiation	[96,117]
miR-107	Downregulated	Tumor suppressor. Involved in differentiation of neuronal cells; interacts with dicer to control the biogenesis of miR-9	[32,118]
miR-124-2-3p	Downregulated	Tumor suppressor. Involved in neuronal identity; regulates adult neurogenesis	[63,119]
miR-145-5p	Downregulated	Tumor suppressor. Crucial for fate determination of neurons	[120,121]
miR-183	Downregulated	Tumor suppressor. Regulates sensory neurons	[74,122]
miR-184	Downregulated	Tumor suppressor. Involved in neural stem cell proliferation and differentiation	[85,123]
miR-193b-3p	Downregulated	Tumor suppressor	[124]
miR-200b-3p	Downregulated	Tumor suppressor. Controls postnatal forebrain neurogenesis	[32,125]
miR-202	Downregulated	Tumor suppressor	[33]
miR-204	Downregulated	Tumor suppressor. Involved in adult somatic stem cell maintenance	[97,126]
miR-335-3p	Downregulated	Tumor suppressor. Implicated in self-renewal of NSCs via inhibition of the p53 signaling pathway	[127,128]
miR-380-5p	Upregulated	Oncogenic	[129]
miR-421	Upregulated	Oncogenic. Involved in NSC self-renewal via the PINK1/HDAC3/FOXO3 axis	[89,130]
miR-449	Downregulated	Tumor suppressor. Essential for brain development; a key regulator of mitotic spindle orientation during neurogenesis	[131,132]
miR-488-5p	Downregulated	Tumor suppressor	[29]
miR-497	Downregulated	Tumor suppressor	[133]
miR-542-3p	Downregulated	Tumor suppressor. Involved in neural development and astrogliogenesis differentiation	[29,134]
miR-542-5p	Downregulated	Tumor suppressor. Involved in neural development and astrogliogenesis differentiation	[29,134]
miR-628-3p	Downregulated	Tumor suppressor	[135]

## Data Availability

Not applicable.
